# Patient involvement in healthcare professional practice – a question about knowledge

**DOI:** 10.1080/22423982.2017.1403258

**Published:** 2017-11-20

**Authors:** Tine Aagaard

**Affiliations:** ^a^ Institute for Nursing and Health Sciences, Ilisimatusarfik/University of Greenland, Nuuk, Greenland

**Keywords:** Patient involvement, patient perspective, chronic illness, everyday life, qualitative research, Greenland

## Abstract

The concept of patient involvement is ambiguous and contested in the healthcare systems in Western Europe and North America. Current research indicates that patients only feel moderately involved in their treatment and care. This article builds on a study of chronically ill patients’ perspectives on healthcare practice in Greenland. It discusses the significance of including in healthcare practice knowledge of patients’ everyday lives with illness and their own views on their situations. Research was qualitative and ethnographic. Participants were followed with participant observations and qualitative interviews for 2.5 years during hospital stay in the capital Nuuk and in their homes in towns and settlements during 2010–2013. Results show that patients are concerned about how to manage their life with illness on a daily basis. Their everyday life activities demonstrate the resources they have to live with illness. However, procedures for healthcare practice concentrate on treatment of the physical disease. Knowledge about psychosocial needs for care and rehabilitation tend to be excluded. The study points to potential for improving professional practice through healthcare professionals’ active investigation of patients’ everyday lives and values, integration of this knowledge into their professional practice and developing structures for this kind of involvement.

## Introduction

Within the last three to four decades, patient involvement in healthcare practice has been placed on the agenda in most Western European and North American countries. Simultaneously, disease and treatment patterns have changed. Large proportions of populations now live longer as a result of improved screening and treatment. The desire to involve patients in their treatment has many sources: Human rights, ethical demands, patients’ legal rights, improvement of professional practice, economic calculations, and more.

However, government reports and qualitative research show that patients feel involved in healthcare practice only to a moderate degree [1–4]. The Danish Government’s latest health strategy from 2014 concedes that the last 20 years of quality improvement, through standardisation and documentation of professional practice, has created a vast bureaucracy that hinders collaborative decision-making between patients and professionals. As a precondition for good quality healthcare practice, new Danish programs aim to reduce levels of bureaucracy in order to restore time for patient-care provider relations [5]. However, the concept of patient involvement is ambiguous. In practice, patient involvement ranges from professionals informing and guiding patients about disease and treatment, to involving patient representatives in the development of procedures of healthcare practice [1,6–8]. In regards to restoring time for the patient-provider relations, one could ask how exactly patient involvement may contribute to quality? What exactly should go on in the relations between patients and professionals? Another question could be whether time is the most important factor for the involvement of patients in professional practice?

Patient involvement has been investigated from a range of theoretical and methodological standpoints. At one end of the continuum are intervention studies that examine tools for patient involvement in professional decisions and review articles about the same. This kind of research is most often concerned with patients’ views of medical treatment [9–11]. The research represents a *disease-oriented* approach to patient involvement [12]. At the other end of the continuum are qualitative studies, often with an ethnographic approach. These studies represent a wider understanding of patient involvement, and often emphasise opportunities given to patients to handle their disease in everyday life and the capacity of the healthcare system to support them [2,3,7,8,13,14]. This research is oriented towards patients’ life situations, i.e. it is *situation-oriented* [12]. However, upon closer examination, much of the situation-oriented research proves to take departure in treatment practice rather than in the patients’ situations. The patients’ everyday lives with chronic illness tend to be viewed through a professional lens, excluding the subjective perspectives of the patients.

This paper draws on the findings from a study of the everyday life of chronically ill patients and their perspectives on healthcare practice in Greenland, which pointed to some common issues related to patient involvement [15]. The study is described here.

## Methodology

### Theoretical approach

All methodologies spring from theoretical reflections on epistemology. Therefore, the theoretical framework of the study is sketched out below.

The concept “Conduct of everyday life” is part of a social-psychological framework developed by German and Danish psychologists starting in the 1960s [16–18]. “Conduct of everyday life” is a way to conceptualise the active process involved in handling one’s life in dialectic interaction with available resources, the conditions at hand and engagement in different social contexts. Each of us organises our everyday activities in a way that is relevant and meaningful from our perspective, taking into consideration time and relationships within and across several contexts such as home, work, leisure-time activities, hospital stays, etc. Seen in this way, illness is an event that erupts into a person’s ongoing life. The central endeavour, however, continues to be to make this life – now with illness – hang together. As a theoretical concept employed within healthcare practice, “Conduct of everyday life” directs the focus radically away from professional practice and into the life contexts of the patients.

Within this theoretical framework, healthcare is seen as a common welfare good, a common practice in which everybody is a participant, both the users of the healthcare institutions and the healthcare professionals. Everybody participates in a dynamic process from her or his location and position and with her or his personal perspectives on shared practices [17,18]. This approach to patient participation conceptualises the fact that patients act in relation to both the professionals and other participants and to structural conditions and, hereby, co-create the contexts they participate in. It emphasises that patients are not just objects of professional interventions.

These reflections on patient involvement led to the following research question: Which opportunities and problems are laid down in the relations between, on the one hand, cultural-historical structural and institutional conditions for providing professional support to patients in their everyday lives with illness and, on the other hand, patients’ historical and cultural preconditions for managing the everyday life with illness?

Operational questions were:

1. What are the patients’ contexts in everyday life?
2. How does the illness affect their everyday life?
3. How do patients handle the situation with illness in relation to their life conditions?
4. How do patients handle the health professional initiatives?

### Design

The study was approved by the Research Ethics Board for Health Research in Greenland. It was conducted between 2010 and 2013 as ethnographic fieldwork on an in-patient ward at the national hospital in Nuuk, Queen Ingrid’s Hospital (QIH), in local healthcare centres and nursing stations in small towns and settlements and in the homes of patients in towns, settlements and the capital Nuuk. The purpose of following patients over time and in space was to get insight into their various contexts, how the contexts were related to each other and how the patients managed the contexts of their everyday lives. The methods used in the empirical research were participant observations, in-depth individual and couple-interviews, focus-group interviews and document analysis.

### Participants

Thirteen patients were interviewed during hospital stay in QIH (see Table 1). Inclusion criteria were: patients suffering from chronic illness, from both rural and urban areas. In three cases, patients’ life partners participated in the interviews (couple-interviews). Among the 13 patients interviewed on the hospital ward, five patients were interviewed in their homes 1–3 times within 12 months after discharge from hospital.^1^ ^1^The decisions about which patients to follow up with interviews at home and how many times were partly dictated by logistic conditions. Greenland is a huge country with a small population of 56,000 people. Towns and settlements are scattered along the more than 2,000 km inhabited coastline and transportation is time-consuming and expensive. It would have been relevant, but not possible, to follow up on all the patients.

**Table 1.** Patients in individual and partner interviews.

Gender and age	Residence	Work	Disease
Female; 49 years	Town	Unskilled worker and housewife	Cancer
Female; 65 years	Town	Unskilled worker and housewife, retired	Radiation damages after treatment for cancer
Male; 55 years	Nuuk	Skilled worker, later on early retirement	Heart disease and diabetes
Female; 50 years	Nuuk	Unskilled worker	Apoplexy
Male; 47 years	Town	Higher education, leader in the public sector	Reumatism
Female; 53 years	Town	Higher education, leader in the public sector	Neurological disease
Male; 74 years	Town	Unskilled worker and selfemployed, retired.	Kidney disease
Female; 48 years	Town	Higher education, leader in the public sector	Cancer
Female; 50 years	Settlement	Skilled worker	Benign tumour
Female; 66 years	Settlement	Unskilled worker and housewife, now retired	Cancer
Female; 54 years	Town	Higher education, leader in the public sector	Cancer
Male; 57 years	Town	Unskilled worker	Heart disease
Female; 51 years	Town	Self-employed tradeswoman	Cancer

### Participant observations

Initially, participant observations were conducted on the hospital ward for 30 workdays and evenings spread evenly over a period of 10 months. Field notes were written concurrently and were reflected on and extended during the entire research process. The purpose was to get insight into the organisational conditions for being a patient and how patients, relatives and professionals operated within these conditions.

### Individual interviews with patients

Participant observations were followed up by interviews with 13 patients on the ward and in their homes. The interviews lasted 1–3.5 hours, with an average of 1.5 hours; they were, with the permission of the interviewees, audio-recorded and transcribed verbatim. According to the research questions, the purpose of the interviews was to gain insight into: (1) the patients’ contexts in everyday life, (2) how the illness affected them, (3) how they handled the situation with illness in relation to their life conditions and (4) how they handled the health professionals’ initiatives. These research questions gave direction to the interview guide, which, moreover, was left open to what the interviewees found important to tell. The focus was on the individual patient’s concrete actions in everyday life and her/his reflections on and subjective understanding of them [19].

### Couple-interviews

These were carried out the same way as the individual interviews. The life partners participated, because both partners wanted it. In all three cases, the participation of the life partner proved very productive because of the dynamics between the partners. They did not always agree with each other on the issues in the interview and this produced knowledge about different meanings of life conditions for persons with different preconditions. Likewise, the different perspectives on the partners’ common situation inspired to further reflection during the interview. Sometimes, the healthy partner held back, not to hurt the ill partner, but this was not dominating in the interviews.

### Focus group interviews

With a view to generating additional knowledge about cultural practices, two focus group interviews were conducted [20,21], one in Nuuk and one in a settlement, which included four and six participants, respectively (see Tables 2 and 3).

**Table 2.** Patients in focus group interview in Nuuk.

Gender and age	Work	Disease
Female; 60 years	Unskilled worker, now retired	Chronical lung disease
Male; 45 years	Skilled worker	Cancer
Female; 58 years	Unskilled worker	Cancer
Female; 42 years	Leader in the public sector	Cancer

**Table 3.** Patients in focus group interview in a settlement.

Gender and age	Work	Disease
Female; 60 years	Work position unknown, retired	Diabetes
Male; 62 years	Unskilled worker, early retirement	Apoplexy
Female; 60 years	Housewife, married to the patient mentioned above	—
Female; 73 years	Leader in the public sector, retired	Cancer
Male; 55 years	Work position unknown, early retirement	Apoplexy
Male; 54 years	Fisherman and hunter, early retirement	Cancer

### Individual interviews with healthcare professionals

To learn about professional practice as a structural and relational condition for the patients’ conduct of everyday life, interviews were also conducted with 13 healthcare professionals. They all worked at QIH or at regional hospitals, healthcare centres or nursing stations in towns and settlements, where the interviewees received treatment.

### Written materials

These included institutional documents like health strategies, clinical guidelines, medical records, patient-information and more. Furthermore, three patients’ medical records, comprising doctors’ and nurses’ records from QIH, Copenhagen University Hospital, Denmark and Greenlandic regional hospitals, healthcare centres and nursing stations were included, with the permission of the patients and the relevant hospital authorities.

### Analysis

The purpose of the analysis was to reveal (1) the connections between institutional practice and the patients’ conduct of everyday life with health-related problems, (2) how the different practices affect each other and (3) what possibilities and problems they hold, compared to the first research question. The patients’ and relatives’ interview material was structured according to their contexts in everyday life, such as home, work, leisure-time activities and hospitalisation. The material from the interviews was supplemented by material from the participant observations, the focus-group interviews, the interviews with the healthcare professionals and the documents mentioned earlier.

The structured material was analysed based on the research questions. The analytic focus was on:

1. The patients’ life conditions (conditions), including healthcare services. Institutional and individual conditions were related to discourses of disease and health and to different cultural life modes in Greenland.
2. How the patients themselves perceived the conditions (meanings).
3. How this either held back or facilitated their possibilities for managing their life with illness in ways that gave meaning for them (reasons) (see Table 4).

**Table 4.** Analytic focus: conditions, meanings and reasons.

Conditions	General societal conditions: Societal and institutional conceptions of knowledge and health Political health strategies Administrative structures Understandings of health among the public at largeIndividual conditions in everyday life: Significant relations, work and other activities Changes as a consequence of the disease Institutional offers of treatment and care
Meanings	The meaning of the conditions for the individual person: Possibilities and limitations of action provided by the conditions Personal perceptions of the conditions
Reasons	Forms of conduct of everyday life as a consequence of the conditions and their personal meanings for the individual

The results were organised as an accumulation and discussion of points from the analyses with reference to the original research question concerning the relations between, on the one hand, the conditions for professional support and, on the other hand, patients’ preconditions. The “themes” were (1) the structural conditions of healthcare practice, (2) patients’ experiences of existing healthcare provisions like treatment, care and rehabilitation and the way this is provided in professional practice in hospital and (3) patients’ subjective handlings of everyday life with illness and what resources they hold.

Practically, this was done by the reading through of the interviews, the participant observation notes and institutional documents time and time again, in order to identify the conditions, their meanings for the individual patient and, consequently, her or his reasons for handling the everyday life with illness. Electronic coding was not used, since the aim of the research was to get insight into the hanging together of contexts. Thus, it makes no sense to isolate interviewee-statements from the contexts in which they were put forward. The analysis-method described here is complex and time-consuming (the method is thoroughly described in Aagaard [22]). No doubt, some meanings have got lost. Likewise, not all themes of relevance for the topic of the research have been possible to deal with. However, this is a condition for all qualitative research. The researcher will always interpret the material, also the selection of material for analysis.

In what follows, some central analytical points from the analysis are presented.

## Results

### Structural conditions for healthcare practice in hospital

One of the conditions for patients’ possibilities for managing their everyday lives with illness is the professional support from the healthcare system. The study of professional support shows that interventions and care plans are often narrowly connected to treatment of the physical condition: Admittance interviews are concerned about the level of physical functioning, medicine, physical aids, etc.; procedures for professional actions are concentrated on examinations of the body, like laboratory tests and clinical observations, and instrumental treatment of the disease; professional conversations with the patients are mostly limited to information from professionals to patients about disease and treatment, and professional discussions about patients’ problems seldom involve the patients themselves. There are few or no procedures for psychosocial interventions like exploring the patients’ concerns about the consequences of the disease and treatment in their everyday lives. This kind of communication between patients and professionals is carried out only arbitrarily in the care work. These results are sustained by research and government reports in Denmark [23].

The study shows that, when the patients’ experiences of health professional practice and how they handle their disease in everyday life are explored, unseen problems and possibilities in healthcare practice are revealed. This concerns treatment as well as care and rehabilitation. In what follows, some examples from the study are given.

### Experiences of treatment

The choice of treatment has consequences for patients’ everyday lives. A one-sided focus on treatment of disease when a person is admitted to hospital coupled with a lack of professional insight into the impact of treatment on patients’ comprehensive life contexts has consequences. This is partly recognised in current healthcare practice when the professionals are concerned about informing the patients about their disease [24]. Knowledge about the disease and how it might develop can help patients take their measures and try to plan their future.

An example from this study is a woman who lived with kidney disease. She and her husband lived in a small town in northern Greenland, with limited possibilities of treatment. Therefore, when complications to the disease arose, the woman was often hospitalised in Nuuk or even in Denmark. This caused long periods of separation from her home and family. For years, the couple had been bewildered about what was happening to her body. They never knew when a complication would strike. They “took one day at a time”, as they said. After some years the topic came up during a hospital stay. Thorough information from the doctors and nurses about the reasons for the complications and possible disease trajectories allowed the couple to plan their future life on the grounds of knowledge of how the disease might develop and consider moving to Nuuk or Denmark to be close to a specialised hospital. Unhappily, due to other problems, the question was not solved before the woman died.

However, the professional information to the couple was only given by accident. Moreover, information from doctors and nurses is seldom sufficient to allow patients to manage their everyday life with illness. This goes, for example, for critically ill patients’ possibilities for planning the last part of their lives, which is illustrated below.

A woman with breast cancer that had metastasised was being treated with chemotherapy every 3 weeks at QIH in Nuuk. Every time she was to receive the treatment she was hospitalised for 3–4 days because she lived in a town several hours from Nuuk by ship or flight.^2^ ^2^There are no roads between towns and settlements in Greenland. All transportation is done by ship, aeroplane or helicopter. The disease developed quickly and the prognosis was bad. The woman was in her forties, was married and had a young daughter. During what was to be her final hospital stay before she died, treatment complications arose. The doctors made various time-consuming experiments to try to solve the problem. This drew out the woman’s hospital stay in Nuuk for several weeks – a period of time she could have spent at home with her loved ones. The doctors made great efforts to help the woman from their perspective, but the narrow focus on life-prolonging treatment and the fact that the treatment was not related to the woman’s life situation had significant detrimental consequences for her end of life quality.

### Experiences of care

Healthcare practice’s orientation towards disease and treatment also goes for the care provided by nurses and healthcare assistants. Obviously, the healthcare professionals spend most of their time executing the procedures for the disease-oriented practice. However, the disease-orientation is also expressed in healthcare professional’s conceptions of psychosocial reactions patients have to their situation with illness. This also appears in the example about the women with breast cancer described above. The woman was an articulate and resourceful person. She did not want anybody to pity her. Frustrated about her prolonged stay in the hospital and the separation from her family, instead of telling the nurses about her concerns she scolded them for being inefficient. On staff meetings the nurses interpreted her anger as a crisis reaction [25], something that she had to go through. This interpretation was based on their academic knowledge. However, this knowledge did not enable them to understand the reasons for the woman’s anger other than a general crisis reaction related to the disease. They did not relate the woman’s reaction to her individual life situation by gaining insight in her speculations about when to go home. Therefore, they could not support her, for example by influencing the doctors’ decisions. The healthcare personnel assessed the woman’s reactions from their own professional point of view and did not relate them to the problems in her everyday life situation or to her own perspective on her needs and wishes – with the above-mentioned consequences for her end of life quality.

### Experiences of rehabilitation

Many patients experience a lack of professional attention to their needs and future prospects for their lives after hospitalisation. They perceive healthcare professionals as focusing on the immediate situation and its constraints. Often, patients feel held back in their recovery and in developing a new way of conducting their everyday life with illness. Many patients feel that their own motivation and efforts are not encouraged. An example is a middle-aged woman who suffered an apoplexy, resulting in paralysis and disruptions of her cognitive capacities. The woman engaged in training to revive her ability to walk. She had a strong motivation, namely to return as quickly as possible to her work and her family life. The healthcare professionals, on the other hand, regarded the woman’s goals in rebuilding her capacities as unrealistic. They saw it as a professional task to confront the woman with her shortcomings, considering that she should not be disappointed when her recovery proved unsuccessful. They did not know the everyday life reasons for the woman’s haste. Thus, instead of engaging in a dialogue with her about a suitable way to reach her goals and maybe revise them and sustaining her motivation, the professionals actually discouraged her. Through her own commitment, the woman managed to regain her ability to walk, but she did not find meaningful ways to handle her everyday life with family activities and occupations after discharge from hospital.

### Resources in everyday life and hospital stay

The study made it very clear that hospital stays are only part of the patients’ ongoing everyday lives. During hospitalisation the patients were concerned about their future conduct of everyday life. Likewise, they tried to conduct their everyday lives in hospital in ways they found meaningful. For example, many patients found support in each other’s company, spending their time in a meaningful way instead of just waiting for ward rounds, tests and treatments. They talked to their fellow patients, helped each other practically, went for walks in the surrounding nature, attended social and cultural events in town, met around story-telling, singing and listening to music and more. Moreover, the interviewees in the study expressed that these activities often enabled them to better manage life after discharge, for example because they had strengthened their physical form, got inspiration about new ways to tackle problems related to illness or developed new perspectives on future opportunities. The participant observation part of the study showed that these rehabilitative and health promotional activities took place independently of the professionals’ practice. However, since the activities were not recognised by the professionals as related to disease and treatment, it seemed arbitrary whether the activities were facilitated or hindered by the healthcare professionals. In this way, the study shows that patients possess many resources, which could contribute to the improvement of professional practice in hospital. However, the disease-orientation of professional practice tends to make the professionals blind to the patients’ resources, because the resources express themselves in everyday activities, which are regarded as irrelevant to treatment of disease. The patients’ knowledge about their own lives and values is not regarded as relevant knowledge for professional practice.

## Discussion

### Knowledge and quality improvement

In connecting patients’ conduct of everyday life to healthcare practice in hospital, the study has pointed out problems in improving the quality of professional practice. Since patients’ knowledge is often excluded from professional decision-making, this knowledge cannot inform and qualify professional practice. This affects the quality of healthcare practice negatively.

The results of the study can be related to the research of the American psychologist and evaluation researcher Thomas Schwandt [26]. Schwandt has shown how healthcare practice is shaped by standardised “objective” measurement and documentation procedures and parameters in an attempt to create scientific knowledge and “evidence-based practice”. Schwandt emphasises that practice is much more complex than this kind of evaluation practice would suggest. He criticises this approach to quality for being instrumental and rationalistic and for overlooking that treatment and care are provided by thinking and acting practitioners. When healthcare practitioners make decisions they choose when and what scientific knowledge is relevant and they use the knowledge in combination with knowledge about the patients’ needs and wishes, institutional resources, ethical considerations and more. That is to say, there are several forms of knowledge at play when decisions are made in healthcare practice: Scientific knowledge, professional theoretical and experiential knowledge and the patients’ perspectives and knowledge about their life [26]. Furthermore, one could add that knowledge is developed under certain circumstances and is not the expression of a universal truth. What is right to do in practice depends on the concrete situation and its’ circumstances. It can never be a direct execution of scientific knowledge. This is exactly what is documented in the study reported in this article. This conception of knowledge as distributed between different participants with different positions and perspectives in practice indicates that an evidence-based healthcare practice must acknowledge all forms of knowledge. There is a clear and urgent need for more research into patients’ and professionals’ perspectives in order to evidence-base these forms of knowledge and improve patient experiences and outcomes.

### Knowledge and patients’ experiences

In much humanistic healthcare research, patients’ perspectives are regarded as subjectivist and irrational, because they are related to individual emotions. In this kind of research, the reason why patients’ perspectives should be involved in healthcare practice is first and foremost ethical, a way of making the patients feel equal with the professionals and making them have a general feeling of comfort and wellness [27–29]. The patients’ perspectives are not regarded as holding substantial knowledge of value for the forming of concrete professional interventions. The success of the interventions is merely seen as a question of transferring professional knowledge about disease and treatment to the patients, and in this way enabling them to manage the disease. This can cause disagreements and conflicts between patients and professionals. Accordingly, many professionals perceive patient involvement as an extra burden in their work [15, p.125–127].

Contrary to this hierarchical conception of knowledge, the Danish philosopher Keld Thorgaard [30] points to the fact that peoples’ ways of handling their lives are motivated by the social contexts they are part of and, therefore, patients’ perspectives give evidence of problems and possibilities in institutional and societal structures. This concept of knowledge points to the significance of combining professional knowledge with the patients’ knowledge. The two different forms of knowledge supplement each other; both are necessary to make the patients’ lives with illness hang together [30]. The findings, reported in this article, clearly show that sub-optimal, unfavourable care is the outcome when patients’ knowledge is excluded from professional decision-making. The findings even show that professional practice can get inspiration for improvement by actively exploring patients’ conduct of everyday life during hospital stay. When this is done, patients’ perspectives are a contribution to professional knowledge, not just irrational feelings that should be dealt with for ethical reasons; patients’ knowledge about their life conditions and life expectations inform professional practice about how to take action in concrete cases. This is not to say that patients are always right. Patients can develop their views of healthcare practice, based on limited knowledge. They also need the professionals’ knowledge. However, this does not legitimise professional knowledge trumping patient knowledge. The two parties have different forms of knowledge that supplement each other. Humanistic health research can contribute to evidence-basing the under-exposed patient-perspectives by describing patients’ conduct of everyday life from the patients’ point of view and relating them to institutional and societal structures and practice.

## Conclusions

The study reported in this article shows that treatment and care in hospital and the patients’ possibilities for handling their disease in everyday life are closely connected. Moreover, the examples from the study show that the healthcare professionals, who work hard on giving the patients the best possible treatment and care during hospital stay, are subjected to procedures and theoretical tools that can actually hinder successful treatment giving a positive influence to the patients’ everyday lives, because the patients’ knowledge of their everyday lives is not included in practice.

The study has shown two major barriers for involving patients in their treatment and care as participants with knowledge and perspectives equally important as the professionals: (1) The disease-oriented structures of healthcare practice and (2) an understanding of professional knowledge as more authoritative and valuable than that of the patients and relatives.

To improve professional practice, patients’ knowledge and perspectives must be included, along with professional clinical and scientific knowledge. This requires two preconditions:

1. Structures and procedures for including patients’ perspectives. This could be healthcare provider–patient dialogues that include the patients’ own expressions of their needs and wishes, and the professionals’ active exploration of the patients’ conditions for managing their everyday lives with illness.
2. A conceptualisation of professional practice that includes the patients and relatives as participants with valuable knowledge and perspectives, not as substitutes for professional knowledge, but as necessary supplements.

These preconditions express a view of patient participation that differs from the situation-oriented approaches mentioned in the beginning of this article, in that they are also *subject-oriented*.

Humanistic health research in patients’ perspectives can contribute to evidence-basing patients’ knowledge. Likewise, humanistic health research in professionals’ perspectives can contribute to revealing barriers and possibilities in the healthcare system for involving the patients in their treatment, care and rehabilitation.
